# Fangchinoline Inhibits Human Immunodeficiency Virus Type 1 Replication by Interfering with gp160 Proteolytic Processing

**DOI:** 10.1371/journal.pone.0039225

**Published:** 2012-06-13

**Authors:** Zhitao Wan, Yimei Lu, Qingjiao Liao, Yang Wu, Xulin Chen

**Affiliations:** State Key Laboratory of Virology, Wuhan Institute of Virology, Chinese Academy of Sciences, Wuhan, Hubei, People's Republic of China; Lady Davis Institute for Medical Research, Canada

## Abstract

The introduction of highly active antiretroviral therapy has led to a significant reduction in the morbidity and mortality of acquired immunodeficiency syndrome patients. However, the emergence of drug resistance has resulted in the failure of treatments in large numbers of patients and thus necessitates the development of new classes of anti-HIV drugs. In this study, more than 200 plant-derived small-molecule compounds were evaluated in a cell-based HIV-1 antiviral screen, resulting in the identification of a novel HIV-1 inhibitor (fangchinoline). Fangchinoline, a bisbenzylisoquinoline alkaloid isolated from Radix *Stephaniae tetrandrae*, exhibited antiviral activity against HIV-1 laboratory strains NL4-3, LAI and BaL in MT-4 and PM1 cells with a 50% effective concentration ranging from 0.8 to 1.7 µM. Mechanism-of-action studies showed that fangchinoline did not exhibit measurable antiviral activity in TZM-b1 cells but did inhibit the production of infectious virions in HIV-1 cDNA transfected 293T cells, which suggests that the compound targets a late event in infection cycle. Furthermore, the antiviral effect of fangchinoline seems to be HIV-1 enve1ope-dependent, as the production of infectious HIV-1 particles packaged with a heterologous envelope, the vesicular stomatitis virus G glycoprotein, was unaffected by fangchinoline. Western blot analysis of HIV envelope proteins expressed in transfected 293T cells and in isolated virions showed that fangchinoline inhibited HIV-1 gp160 processing, resulting in reduced envelope glycoprotein incorporation into nascent virions. Collectively, our results demonstrate that fangchinoline inhibits HIV-1 replication by interfering with gp160 proteolytic processing. Fangchinoline may serve as a starting point for developing a new HIV-1 therapeutic approach.

## Introduction

The global epidemic of acquired immunodeficiency syndrome (AIDS) remains one of the most pressing public health emergencies. According to the latest statistics provided by the Joint United Nations Programme on HIV/AIDS (UNAIDS), there were an estimated 33.3 million people living with HIV and 1.8 million deaths due to AIDS in 2009. Since the emergence of HIV, intensive studies have been undertaken to understand the disease and find effective therapies. Currently, highly active antiretroviral therapy (HAART) is considered to be the standard therapy formula and has been proven to be successful in controlling viral replication and disease progression. However, prolonged treatment with combination regimens can be difficult to sustain because of problems with adherence and toxic effects [1], [2]. Furthermore, in a significant portion of treatment-experienced patients, therapeutic options are limited due to the emergence of drug resistance [3]. Moreover, cross-resistance between drugs within a class frequently occurs [4]. Theoretically, the discovery and development of drugs targeting alternative mechanisms in the HIV-1 replication cycle should be the most effective strategy to overcome drug resistance.

The HIV-1 envelope (Env) glycoproteins play an essential role in the viral replication cycle by mediating attachment and fusion of the viral envelope with the host cell membrane [5]. The Env glycoproteins are synthesized as an inactive polyprotein precursor (gp160) on the rough endoplasmic reticulum (RER) and targeted to the endoplasmic reticulum (ER) by its N-terminal signal peptide. Concomitant with translation, the signal peptide is removed, and gp160 is subjected to extensive glycosylation. Monomers of gp160 assemble into trimers and traffic into the Golgi through the constitutive secretory pathway. In the Golgi, gp160 is proteolytically cleaved by cellular furin or furin-like proteases at a highly conserved motif to yield the surface subunit gp120 and the transmembrane subunit gp41 [6]. Following cleavage, gp120 and gp41 remain associated by noncovalent interactions. Thereafter, the gp120/gp41 trimeric complex is transported to the plasma membrane where it is incorporated into the envelope of assembling HIV-1 particles.

Because the proteolytic cleavage of the viral envelope glycoprotein is essential for the production of infectious virions [7], gp160 processing might be an attractive target for anti-HIV-1 intervention [8]. Peptide or small-molecule inhibitors targeting the expression of a functional HIV-1 Env have been previously reported. Peptides derived from the cleavage region of gp160 have been shown to inhibit gp160 processing and suppress productive HIV-1 infection [9], [10], [11], [12], [13]. However, development of such molecules for clinical application will face the challenges associated with developing drugs from peptide-based compounds [14]. Furthermore, because a large number of host proproteins require the host proprotein convertases for maturation and because all proprotein convertases share the consensus (R/K)-*X*-R cleavage sequence [15], these peptide substrate-competitive inhibitors may also inhibit the cleavage of other host protein substrates in the same manner. Small-molecule compounds that disrupt intracellular trafficking or glycosylation can also consequently inhibit gp160 cleavage into its active forms [6], [16], [17], [18]. However, the clinical potential of this class of compounds is limited due to the cell toxicity caused by interfering with constitutive cellular functions. More recently, a small-molecule compound that interferes with gp160 processing resulting in the inhibition infectious-virion production has been described [19]. However, this compound exhibits a poor spectrum of activity against HIV-1 laboratory strains, and its antiviral mechanism requires further elucidation.

Plants remain an important source of new drugs, new leads and new chemical entities. Many compounds of plant origin have been identified that inhibit different stages in the replication cycle of HIV [20], and some of such compounds are currently in clinical trials [20], [21]. Therefore, plant-derived compounds may serve as starting point for the development of anti-HIV drugs. In the present study, we describe that a plant-derived small-molecule compound, fangchinoline (6, 6′, 12-trimethoxy-2, 2′-dimethyl-berbaman-7-ol), inhibits HIV-1 propagation by targeting Env at a late stage of the viral replication cycle. Fangchinoline was identified as a novel anti-HIV-1 agent by a cell-based screen of compounds derived from Chinese herbal remedies. We show here that fangchinoline inhibited HIV replication in MT-4 and PM1 cells but was not active in TZM-b1 cell assays. In a virus production assay, fangchinoline reduced the production of infectious virions in an Env-dependent manner. Further mechanism-of-action studies demonstrated that fangchinoline specifically interferes with gp160 processing and thus reduces the incorporation of Env into nascent viral particles.

## Materials and Methods

### Cell lines, plasmids and viruses

The human embryonic kidney cell line 293T was obtained from American Type Culture Collection (ATCC). MT-4, C8166, PM1 and TZM-b1 cell lines were obtained through the NIH AIDS Research and Reference Reagent Program. TZM-b1 cells and 293T cells were propagated in Dulbecco's modified Eagle's medium (DMEM) (Invitrogen) supplemented with 10% FBS (Gibco), 100 U/mL of penicillin and 0.1 mg/mL of streptomycin. MT-4, PM1 and C8166 cells were grown in RPMI 1640 supplemented with 10% FBS and 10 µg/mL gentamicin. The HIV-1 proviral clones pNL4-3 and pLAI.2, and the env-deficient proviral clone pSG3Δenv were obtained through the AIDS Research and Reference Reagent Program. The vesicular stomatitis virus glycoprotein (VSV-G) expressing plasmid pVpack-VSV-G was purchased from Stratagene. The HIV-1 BaL Env expression plasmid, which was constructed by inserting the Env sequence from HIV-1 BaL into pcDNA3.1, was provided by Dr. Qinxue Hu (Wuhan Institute of Virology, Chinese Academy of Sciences). The HIV-1 strains NL4-3 and LAI were generated by transfecting 293T cells with the corresponding plasmids and were propagated in MT-4 cells. The HIV-1 strain BaL was obtained through the AIDS Research and Reference Reagent Program.

### Antibodies and compounds

Anti-VSV-G tag antibody (ab34774), anti-HIV-1 gp120 (ab21179) antibody and anti-HIV-1 p24 antibody (ab9071) were purchased from Abcam. Anti-beta actin antibody (sc-130301) was purchased from Santa Cruz. All horseradish peroxidase (HRP)–labeled secondary antibodies were obtained from Thermo Scientific.

The compounds isolated from Chinese traditional herbs for the anti-HIV-1 drug screen were purchased from Sichuan Weikeqi Biological Technology Co., Ltd., China. The purity of the compounds was ≥98% by high-performance liquid chromatography (HPLC) analysis. The compound samples were solved in dimethyl sulfoxide (DMSO) at a concentration of 50 mM. The following reference compounds were obtained through the NIH AIDS Research and Reference Reagent Program, Division of AIDS, NIAID, NIH: indinavir sulfate (catalog no. 8145), zidovudine (catalog no. 3485), integrase inhibitor 118-D-24 (catalog no. 9957) and (-) flavopiridol (catalog no. 9925). Dextran sulfate sodium salt (average molecule weight 5,000) was purchased from Sigma-Aldrich.

### *In vitro* anti-HIV-1 assays

The anti-HIV-1 activities of the compounds were evaluated utilizing a variety of assays, including a cytopathic effect (CPE) assay, p24 assay and TZM-b1 assay.

The CPE assay was performed in 96-well black-wall tissue culture plates using MT-4 cells as targets. MT-4 cells were infected with HIV-1 LAI or NL4-3 at a multiplicity of infection (MOI) of 0.1. To each well containing 1×10^4^ MT-4 cells, serial diluted test compounds were added. Cell viability was measured 5 days post-infection using the CellTiter-Glo reagent (Promega) according to the manufacturer's instructions. The luminescent signal was determined using the Envision 2102 Multilabel Reader (Perkin Elmer). The EC_50_ (50% effective concentration) values correspond to compound concentrations that resulted in a 50% reduction in cell death.

In p24 assays, MT-4 or PM1 cells were infected with HIV-1 LAI, NL4-3, or BaL at an MOI of 0.01. Infected cells were plated in 96-well plates at a density of 1×10^4^ per well and serial diluted test compounds were added. On day 4 post-infection, culture supernatants were harvested and treated with Triton X-100. The level of viral replication was determined by an HIV-1 capsid protein (p24) antigen capture enzyme linked immunosorbent assay (ELISA). Compound cytotoxicity was also determined in parallel in mock-infected cells.

The TZB-b1 assay was utilized to determine the inhibitory activity of fangchinoline against early and late events of the HIV-1 replication cycle. TZM-b1 cells contain the HIV primary receptor CD4 and the coreceptors CCR5 and CXCR4, as well as a firefly luciferase reporter gene driven by the HIV promoter. In this assay, TZM-b1 cells were plated 1×10^4^ per well in 96-well tissue culture plates one day before infection. On the day of the experiment, the cell supernatant was removed, and serial diluted compounds in volumes of 100 µL were added. HIV-1 NL4-3 in 100 µL of complete medium was then added to each well to achieve an MOI of 1. At 48 hours post-infection, luciferase activity in the cells was analyzed with the Steady-Glo reagent (Promega).

### Semi-quantitative polymerase chain reaction (PCR) analysis of intracellular HIV-1 viral DNA and mRNA

MT-4 cells were grown in 24-well plates and infected with NL4-3 strain at a MOI of 0.02, and then test compounds were added to desired concentrations. After incubating for 3 days, genomic DNA and mRNA from the infected cells were isolated using a Genomic DNA Mini Preparation Kit (Beyotime, China) and TRIzol reagent (Invitrogen Life Technologies), respectively. The Gag region, representing total viral DNA, was amplified with previously described primers [22]. A nested PCR was used for the amplification of integrated proviral DNA, as previously described with minor modification [23]. To determine viral mRNA expression levels, 1 µg of RNA was treated with RQ1 RNase-Free DNase (Promega) and reverse transcribed using M-MLV Reverse Transcriptase (Promega) and random primers (Promega). An aliquot of cDNA was used as a template for amplification of the HIV-1 Gag region as described elsewhere [22]. As DNA and RNA input controls, genomic DNA and cDNA was subjected to GAPDH amplification using the primers 5′-GAAGGTGAAGGTCGGAGTC-3′ and 5′-GAAGATGGTGATGGGATTTC-3′. All of the PCR products were visualized on an ultraviolet (UV) transilluminator in 2% agarose gels stained with ethidium bromide. In preliminary experiments, the exponential range of the PCR amplification curve was determined for all of the PCR products by varying the amount of input DNA and the number of PCR cycles. Based on these experiments, appropriate conditions were chosen to perform semi-quantitative PCRs.

### Virus production/infectivity assays

293T cells (90% confluent) grown in 24-well plates were transfected with the HIV-1 infectious clone pNL4-3 or co-transfected with env-deficient proviral clone pSG3^Δenv^ and an envelope expression plasmid, either HIV-1 BaL Env expression plasmid or pVpack-VSV-G, using the FuGENE HD transfection reagent (Roche). At 3 hours post-transfection, the cell supernatants were removed and fresh medium with test compounds at the indicated concentration were added. At 48 hours after transfection, the virus release, virus infectivity and 293T cell viability were examined. The supernatants of the transfected cells were harvested for the evaluation of virus production and infectivity determination. To evaluate the cytotoxicity of the test compounds on 293T cells, the cells were resuspended in 500 µL of DMEM medium, and 50 µL of the cell mixture was transferred to 96-well plates to determine cell viability using the CellTiter-Glo reagent. To detect virus release, one portion of culture supernatant was treated with Triton X-100 and examined using the p24 ELISA. The infectivity of the virus in the culture supernatants was also determined utilizing the TZM-b1 assay. A total of 20 µL of supernatant in each well was transferred to 96-well plates containing TZM-b1 cells in 180 µL of medium plated one day before. The luminescence was determined 48 hours post-infection using the Steady-Glo reagent.

### Syncytium formation assays

MT-4 cells were infected with HIV-1 NL4-3 at a high MOI of 1. After incubating for 24 hours, the cells were washed and incubated with 1 µM indinavir (IDV) alone or with 1 µM IDV plus 2.5 µM fangchinoline for an additional 24 hours. Thereafter, MT-4 cells were cultured with an equal number of the C8166 cells in a culture plate in the presence of the same compounds in duplicate. After 24 hours of cocultivation, syncytium formation was examined using light microscopy.

### Western blot analysis

293T cells were transfected with pNL4-3 as described above. At 3 hours post-transfection, the cell supernatant was removed and fresh medium with a test compound was added. At 48 hours post-transfection, the supernatants of the transfected cells were first clarified by centrifugation for 5 min at 1,000 g at 4°C. Supernatants were then layered on a 0.25-mL sucrose cushion (20% sucrose/100 mM NaCl/10 mM Tris buffer, pH 7.5) and centrifuged at 4°C and 13,000 rpm (17,000 *g*) for 3 hours to pellet the virions. After centrifugation, the supernatants were removed, and the virus pellets were resuspended in 50 µL SDS polyacrylamide gel electrophoresis (SDS-PAGE) sample buffer and boiled for 10 min. To prepare the cell-derived western blot samples, the transfected cells were washed with phosphate buffered saline (PBS) and lysed with radio immunoprecipitation assay (RIPA) lysis buffer supplemented with complete mini protease inhibitor cocktail (Roche) at 4°C for 1 hour. Proteins from concentrated virions and cell lysates were separated by SDS-PAGE and transferred to polyvinylidene difluoride (PVDF) membranes. After overnight incubation with primary antibodies at 4°C, each blot was probed with horseradish peroxidase-conjugated secondary antibody. Immunoreactive signals were detected with an enhanced chemiluminescence substrate (Thermo) using an AlphaEaseH FC Imaging System (Alpha Innotech Corporation).

### Pulse-chase analysis

Newly synthesized protein was labeled with Click-iT Methabolic Labeling Reagents for Proteins (Invitrogen) according to manufacturer's instructions. 293T cells were transfected with pNL4-3 as described above. At 3 hours post-transfection, the cell supernatant was removed and fresh medium with or without 10 µM fangchinoline was added. After 24 hours, the cells were labeled with 50 µM of AHA (Invitrogen) for 1 hour in methionine-free medium with or without fangchinolie. The medium was then changed with complete medium with or without fangchinolie, and the cells were chased for indicated time. The cells were lysed in the lysis buffer [(50 mM Tris HCl pH 8.0, 1%SDS, and protease inhibitor (Roche)]. AHA incorporated protein was biotinylated using Click-iT Protein Reaction Buffer Kit (Invitrogen) and Biotin Alkyne (Invitrogen). After precipitation and dissolving, biotinylated protein was collected with Dynabeads MyOne Streptavidin T1 (Invitrogen). The purified nascent protein was eluted into SDS-PAGE sample buffer by boiling and examined by Western blot analysis as described above.

## Results

### Screen of compounds derived from Chinese herbal remedies led to the identification of fangchinoline as a novel anti-HIV-1 agent

To identify novel anti-HIV agents, a set of more than 200 compounds isolated from traditional Chinese medicinal herbs was screened utilizing an MT-4 cell CPE assay. In this HIV multiple-cycle replication assay, the activity of the compounds against HIV-1 replication was determined based on the inhibition of virus-induced cytopathogenicity in MT-4 cells. Thus, compounds having anti-HIV activities at any stage of the viral replication cycle can be detected by this assay. Although the model does not allow for high throughput screening, it incorporates all of the HIV-1 targets required for replication and provides an opportunity to identify compounds with novel anti-HIV-1 mechanisms.

**Figure 1:**
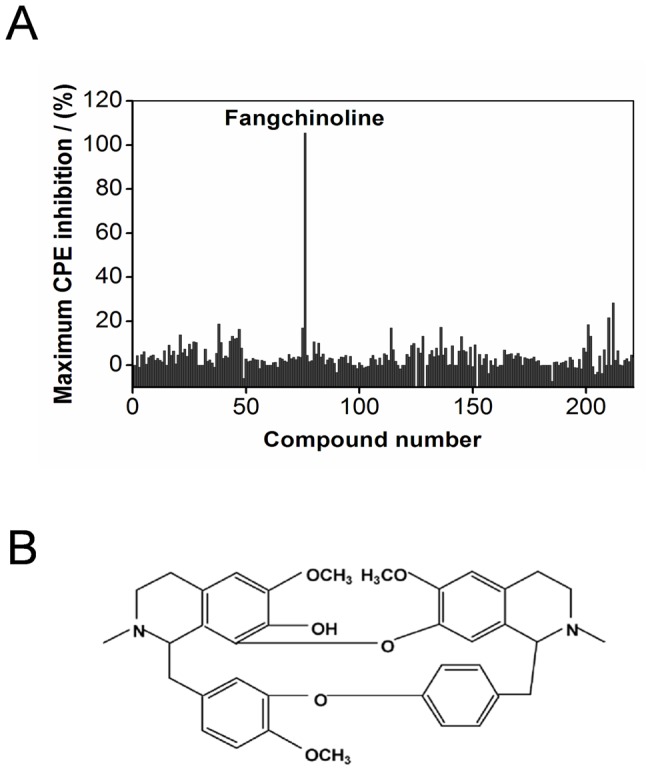
Screen of compounds derived from Chinese herbal remedies led to the identification of fangchinoline as a novel anti-HIV-1 agent. (A) MT-4 cells were infected with HIV-1 LAI at an MOI of 0.1 or mock infected in the presence of serially diluted test compounds. Cell viability was measured 5 days post-infection. The anti-HIV activity of each compound was presented as the maximum inhibition of CPE at various concentrations. (B) The chemical structure of fangchinoline.

All the compounds were tested over a wide range of concentrations using 7 rounds of 3-fold serial dilutions starting at 30 µM. The anti-HIV activity of each compound was presented as the maximum inhibition of virus-induced CPE at various concentrations. Among all the tested compounds, fangchinoline, a bisbenzylisoquinoline alkaloid isolated from Radix *Stephaniae tetrandrae*, showed the most potent antiviral activity against HIV-1 strain LAI (Fig. 1). At the concentration 1.1 µM, fangchinoline completely inhibited the CPE induced by HIV-1 replication.

### *In vitro* anti-HIV-1 activity of fangchinoline

**Figure 2:**
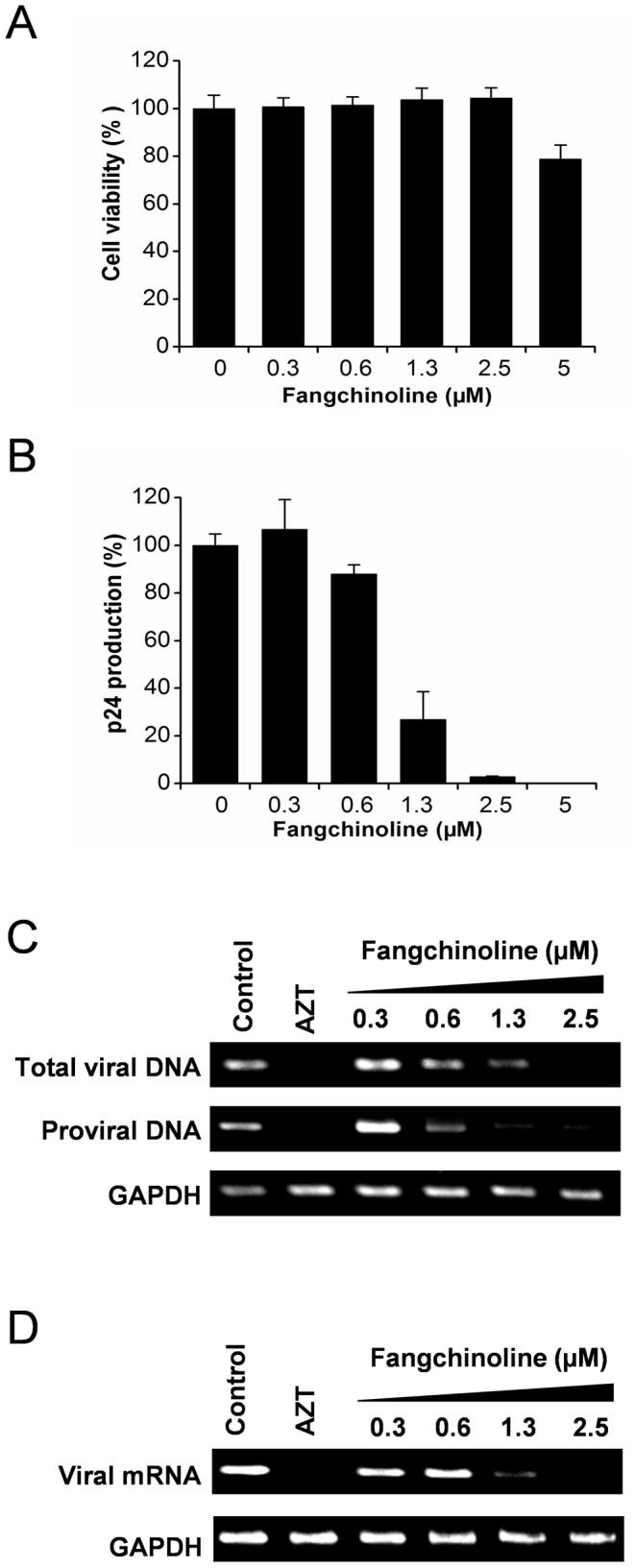
Fangchinoline inhibits HIV-1 NL4-3 replication in MT-4 cells. (A and B) MT-4 cells were infected with HIV-1 NL4-3 at an MOI of 0.02 or mock infected in the presence of serially diluted fangchinoline. On day 4 post-infection, compound cytotoxicity was determined in parallel in mock-infected cells (A), and the level of viral replication in infected cells was determined by p24 antigen capture ELISA (B). The results were presented as the mean values with standard deviations. (C and D) MT-4 cells were infected with HIV-1 NL4-3 at an MOI of 0.02 in the presence of test compounds (AZT, 0.05 µM; fangchinoline, 0.3–2.5 µM), and genomic DNA and mRNA from infected cells were isolated 3 days post-infection. Total viral DNA (C, upper panel), integrated proviral DNA (C, middle panel) were examined by semi-quantitative PCR analysis. Viral mRNA levels were examined by semi-quantitative reverse transcription PCR analysis (D, upper panel). GAPDH was used as both the DNA and RNA input control.

To confirm the anti-HIV-1 activity of fangchinoline in MT-4 cells, p24 assays were performed. NL4-3 infected MT-4 cells were cultured in the presence of various concentrations of fangchinoline, and p24 antigen production was determined by ELISA. To exclude the possibility that the inhibitory effect was due to nonspecific cytotoxicity, cell viability assays were performed in parallel. As shown in Fig. 2B, fangchinoline inhibited p24 antigen expression in a dose-dependent manner at concentrations ranging from 0.6 µM to 2.5 µM, which were below the toxicity threshold (5 µM) for the host cells. At 2.5 µM, fangchinoline reduced p24 antigen expression by 97.2% without obvious toxicity (Fig. 2A and 2B), suggesting the compound specifically inhibited viral replication without alteration of the host metabolism.

To further characterize the effect of fangchinoline on HIV-1 replication, viral DNA and mRNA synthesis were determined by semi-quantitative PCR assays. Total DNA and RNA of NL4-3 infected MT-4 cells were extracted and total viral DNA, integrated proviral DNA and viral mRNA were amplified as described in the Materials and Methods. Consistent with the observations in p24 assays, at non-toxic concentrations (0.6–2.5 µM) fangchinoline inhibited total viral DNA, integrated proviral DNA and viral mRNA synthesis in a dose-dependent manner (Fig. 2C and 2D). All these results confirmed the antiviral activity of fangchinoline against HIV-1 NL4-3 in MT-4 cells.

We next determined whether the anti-HIV activity of fangchinoline was specific to the cell type or viral strain. Antiviral activity of fangchinoline against different viral strains in different host cells was determined in p24 assays. As shown in Table 1, fangchinoline exhibited comparable potency against T-tropic strains (NL4-3, LAI) and an M-tropic strain (BaL) with EC_50_ values ranging from 0.8 to 1.7 µM and selective indexes (SIs) from 3.8 to 8.6. Thus, fangchinoline showed relatively weak but substantial antiviral activity against different HIV-1 strains in T cell lines.

**Table 1:** *In vitro* antiviral activities of fangchinoline against different HIV-1 laboratory strains.

Virus strain	Cell line	EC_50_ (µM)^a^	CC_50_ (µM)^b^	SI^c^
HIV-1 NL4-3	MT-4	0.8	6.9	8.6
HIV-1 NL4-3	PM1	1.5	6.4	4.3
HIV-1 LAI	MT-4	1.3	6.9	5.3
HIV-1 BaL	PM1	1.7	6.4	3.8

Antiviral assays were performed as described in Materials and Methods. Values represent average of at least three independent experiments.

a Compound concentration required to reduce the production of p24 antigen by 50%.

b Compound concentration required to reduce mock-infected cell viability by 50%.

c Selective index, ratio of CC_50_/EC_50_.

### Fangchinoline does not act at early stages of the HIV-1 replication cycle

To determine the stage of the replication cycle targeted by fangchinoline, a TZM-b1 assay was performed. TZM-b1 cells are permissive to HIV-1 infection and harbor an integrated copy of the luciferase gene under transcriptional control of the HIV long-terminal repeat sequence [24]. Reporter gene expression is induced by viral Tat protein after infection. This assay monitors the early steps of infection up to the integration of the viral cDNA into the host cell chromosome and expression of the viral gene Tat [25]. Fangchinoline along with a panel of reference compounds with known anti-retroviral mechanisms were evaluated in this assay. For comparison, the anti-HIV-1 activities of these compounds in MT-4 CPE assays were also determined.

As shown in Table 2, the compounds acting at the early stages of the replication cycle, including dextran sulfate (entry inhibitor), zidovudine (AZT, reverse-transcriptase inhibitor), integrase inhibitor 118-D-24 (INI 118-D-24) and flavopiridol (gene expression inhibitor), caused significant reductions in luciferase gene expression with comparable potency as determined in MT-4 CPE assays. However, the protease inhibitor indinavir (IDV), which acts at a late stage after gene expression, did not show inhibitory effects at concentration as high as 5 µM. As observed in the case of indinavir, fangchinoline did not exhibit measurable antiviral activity up to concentrations that were cytotoxic to the host cells, indicating that fangchinoline blocks viral replication at a stage after viral DNA integration and gene expression.

**Table 2:** Anti-HIV activities of test compounds in MT-4 CPE assays and TZM-b1 assays.

Compound	EC_50_ in CPE assays^a^	EC_50_ in TZM-b1 assays^b^	Stage of action
Dextran sulfate	0.3 µg/mL	0.1 µg/mL	Entry
AZT	5.7 nM	4.9 nM	Reverse transcription
INI 118-D-24	13.6 µM	4.0 µM	Integration
Flavopiridol	2.1 nM	2.6 nM	Gene expression
IDV	5.3 nM	>5.0 µM	Maturation
Fangchinoline	0.7 µM	>5.0 µM	

Antiviral assays were performed as described in Materials and Methods. Values represent average of at least three independent experiments.

a Compound concentration required to reduce the virus-induced CPE by 50%.

b Compound concentration required to reduce the luciferase expression in virus infected TZM-b1 cells by 50%.

### Fangchinoline inhibits the production of infectious HIV-1 particles

**Figure 3:**
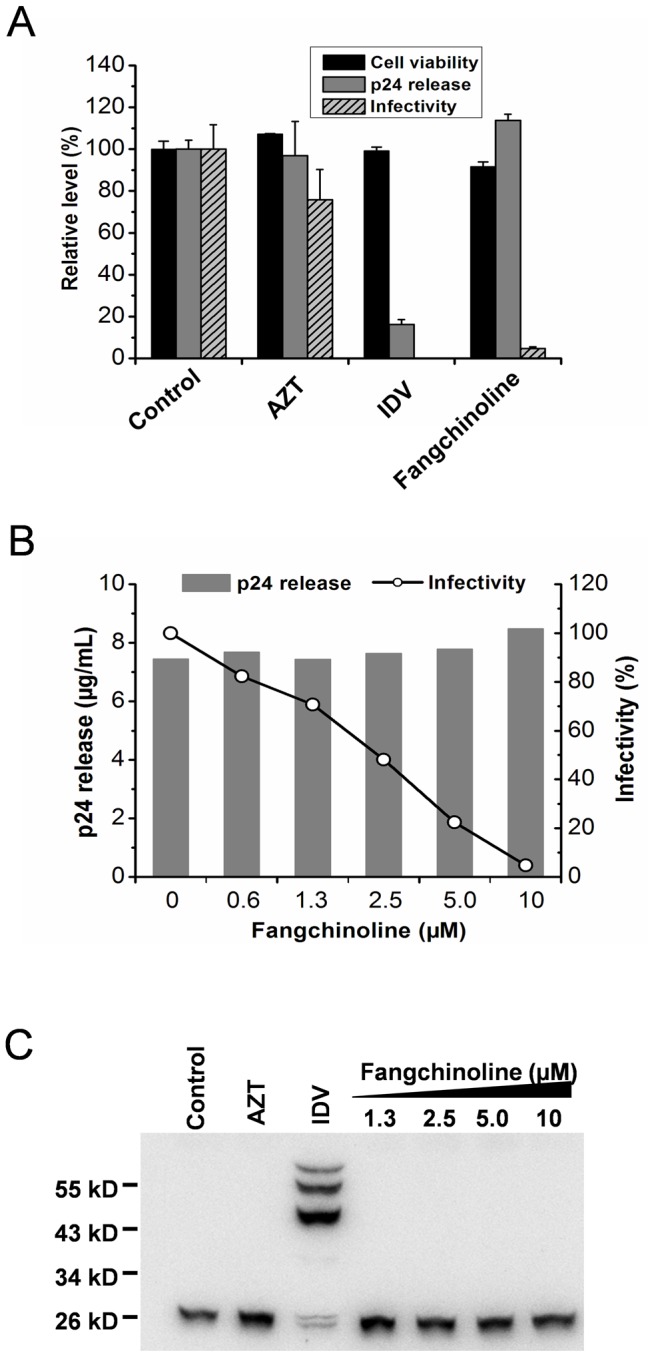
Fangchinoline inhibits the production of infectious HIV-1 particles. (A and B) 293T cells were transfected with the HIV-1 infectious clone pNL4-3. At 3 hours post-transfection, the cell supernatant was removed, and fresh medium with test compounds (AZT, 0.05 µM; IDV, 1 µM; fangchinoline, 0.6–10 µM) was added. At 48 hours after transfection, viral release, viral infectivity and the cell viability of 293T were examined, as described in the Materials and Methods. (C)The Gag protein products derived from virions produced from pNL4-3 transfected 293T cells in the presence of test compounds (AZT, 0.05 µM; IDV, 0.25 µM; fangchinoline, 1.3–10 µM) were examined by Western blot analysis.

To confirm fangchinoline targets a late event in the HIV-1 replication cycle, the effect of fangchinoline on infectious HIV-1 particle production was examined in the virus production/infectivity assay. Infectious virus was produced by transfecting 293T cells with infectious HIV-1 viral cDNA pNL4-3 in the presence or absence of compounds, and the infectivity of the nascent virus was tested utilizing TZM-b1 indicator cells. Although cytotoxicity to MT-4 cells was observed as concentrations exceeded 5 µM, fangchinoline didn't obviously affect the cell viability of 293T cells at 10 µM (Fig. 3A). AZT and IDV were also tested in the assay as early stage and late stage inhibitor controls. As expected, AZT was not active in this assay, while IDV completely inhibited the infectious virus production at the test concentration (Fig. 3A). Of note, fangchinoline dose-dependently inhibited the production of infectious virus with an EC_50_ value of approximate 2.5 µM (Fig. 3B). In contrast, fangchinoline treatment did not affect HIV-1 particle production as the extracellular viral capsid protein levels were comparable with the untreated control (Fig. 3B), which indicates that fangchinoline does reduce the infectivity of nascent particles at a late step of the replication cycle.

Because it was reported that protease inhibitors and maturation inhibitors, which inhibit Gag proteolytic processing, can reduce the infectivity of nascent viruses [26], [27], we further tested whether fangchinoline acts by a similar mechanism. Gag protein products derived from virions produced from pNL4-3 transfected 293T cells in the presence of the test compounds were examined by Western blot analysis, as described in the Materials and Methods. As a positive control, IDV was shown to inhibit the viral protease resulting in the detection of multiple uncleaved Gag precursor proteins. In contrast, Gag processing was not affected in virions produced in the presence of the negative control AZT or fangchinoline (Fig. 3C). These results demonstrated that fangchinoline does not reduce virus infectivity by interfering with HIV-1 virion maturation.

### Fangchinoline specifically reduces the incorporation of HIV-1 Env into nascent virus particles

**Figure 4:**
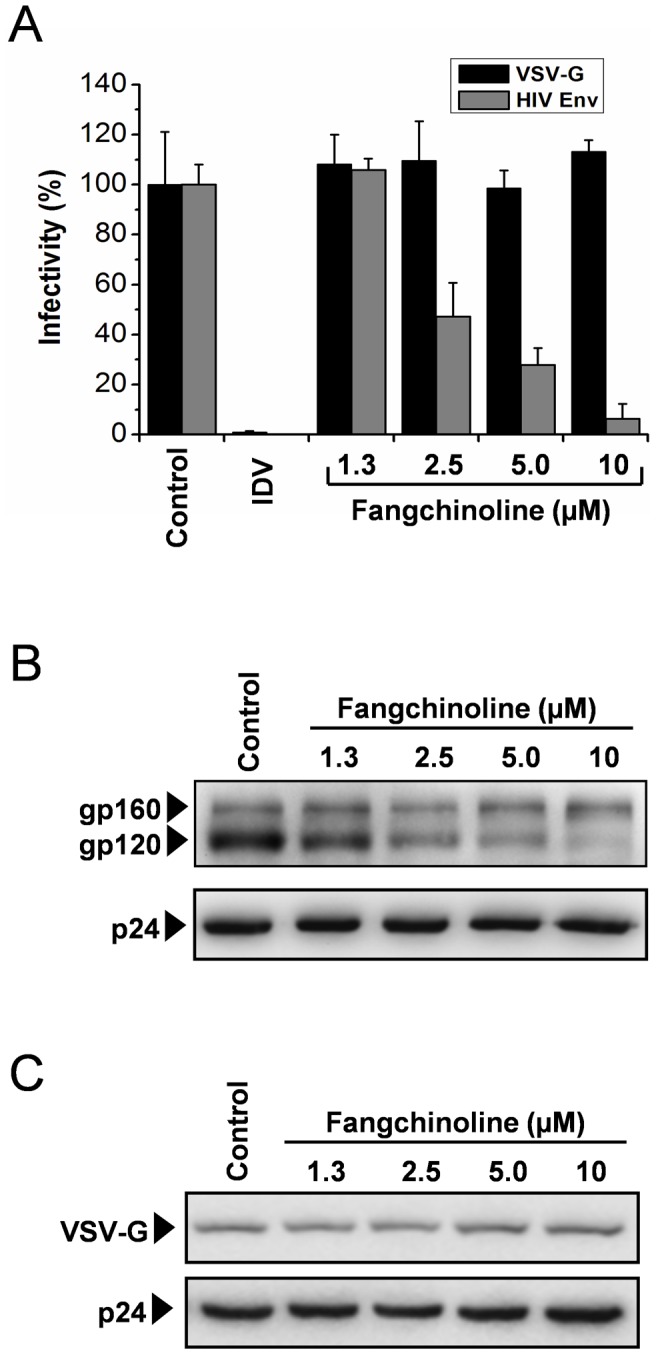
Fangchinoline specifically reduces the incorporation of HIV-1 Env into nascent virus particles. (A) 293T cells were co-transfected with pSG3^ΔEnv^ and an envelope expressing plasmid. At 3 hours post-transfection, the cell supernatant was removed, and fresh medium with test compounds (IDV, 1 µM; fangchinoline, 1.3–10 µM) was added. At 48 hours after transfection, the infectivity of the virions produced by the co-transfected cells was determined in the TZM-b1 assay. (B) 293T cells were transfected with pNL4-3. At 3 hours post-transfection, the cell supernatant was removed, and fresh medium with indicated concentration of fangchinoline was added. At 48 hours after transfection, the content of Env in the HIV-1 particles produced by the pNL4-3 transfected 293T cells was analyzed by Western blot. (C) 293T cells were cotransfected with pSG3^Δenv^ and pVpack-VSV-G. At 48 hours after transfection, the content of VSV-G in the pseudotyped HIV-1 particles produced in the presence or absence of fangchinoline was analyzed by Western blot.

Because the decreased infectivity of HIV-1 particles produced in the presence of fangchinoline could be due to the reduced ability of the virus to enter target cells and because Env plays an essential role in the initiation of infection, we next tested whether fangchinoline inhibits the production of infectious virions in an Env-dependent manner. HIV Gag particles packaged with HIV-1 Env (BaL) or with heterologous envelope (vesicular stomatitis virus G glycoprotein, VSV-G) were prepared by transient transfection in 293T cells and titrated in TZM-b1 indicator cells. As shown in Fig. 4A, the presence of fangchinoline remarkably reduced the infectivity of the particles pseudotyped with wild-type HIV-1 Env. However, fangchinoline treatment did not significantly alter the infectivity of the virus particles packaged with the VSV-G envelope. Unsurprisingly, protease inhibitor IDV was active against both pseudoviruses as it targets Gag particle maturation, which was required by both psuedoviruses (Fig. 4A). These results strongly suggested that fangchinoline specifically targets HIV-1 Env late in viral replication cycle.

At a late stage of HIV-1 replication cycle, Env is trafficked to the cellular surface for incorporation into the assembling virus particles. To determine if fangchinoline affects the incorporation of HIV-1 Env proteins into virions, we next examined the content of Env in the HIV-1 particles produced by 293T cells in the presence of fangchinoline by Western blot analysis. As shown in Fig. 4B, the incorporation gp120 was reduced by fangchinoline in a concentration-dependent manner, whereas the incorporation of gp160 and the p24 levels remained unaffected. In the presence of 10 µM fangchinoline, the incorporation of gp120 was substantially abolished, which is consistent with the observation in the virus production/infectivity assay in which the production of infectious viral particles was reduced by 95.3% (Fig. 3B). On the contrary, the incorporation of VSV-G into pseudotyped HIV-1 particles was not affected by fangchinoline at 1.3–10 µM (Fig. 4C). These data showed that fangchinoline directly or indirectly reduces Env incorporation into HIV-1 particles.

### Fangchinoline reduces functional Env expression by interfering with gp160 processing

**Figure 5:**
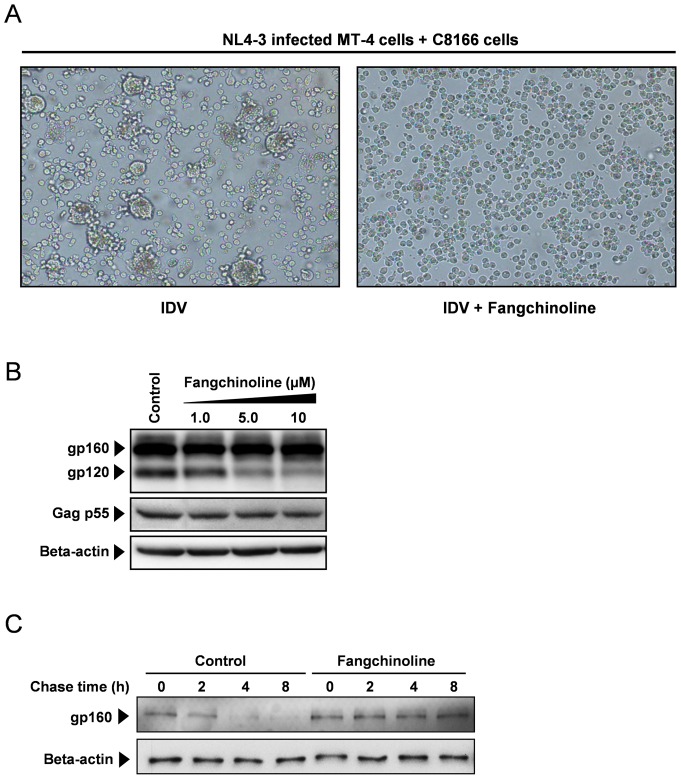
Fangchinoline reduces functional Env expression by interfering with gp160 processing. (A) HIV-1 NL4-3-infected MT-4 cells were pretreated with 1 µM IDV alone (left panel) or 1 µM IDV plus 2.5 µM fangchinoline (right panel) for 24 hours and then cocultured with C8166 cells. After 24 hours of cocultivation, syncytium formation was examined using light microscopy. (B) 293T cells were transfected with pNL4-3 and treated with the indicated concentration of fangchinoline. The Env protein expression in transfected cells was analyzed by Western blot using a polyclonal antibody directed against HIV-1 gp120 48 hours post-transfection. (C) 293T cells were transfected with pNL4-3. At 3 hours post-transfection, the cell supernatant was removed and fresh medium with or without 10 µM fangchinoline was added. After 24 hours, the cells were labeled for 1 h and chased for indicated times in the presence or absence of fangchinoline. After biotinylated, the newly synthesized protein was collected with streptavidin-coupled magnetic beads. Processing of newly synthesized gp160 was examined by Western blot analysis.

Although the actual mechanism of Env incorporation still remains a matter of controversy, all the current postulated models suggest that functional Env presentation on the surface of producer cells is essential [5]. Thus, we next investigated the effect of fangchinoline on the expression of functional Env on the cell surface using a syncytium formation assay. In the assay, functional Env expression on the surface of effector cells induces cell-cell fusion between cocultured cells. NL4-3 infected MT-4 cells were either untreated or treated with 2.5 µM fangchinoline for 24 hours. In addition, 1 µM IDV was added to prevent the production of infectious virions. Then, the cells were cocultured with C8166 cells, which are sensitive to Env-induced syncytium formation. As shown in Fig. 5A, IDV alone did not inhibit the formation of large multinuclear cells. In contrast, in the presence of fangchinoline, giant multinuclear cells were rarely observed. Considering fangchinoline is not active at the early stages of the viral replication cycle, we conclude that fangchinoline treatment reduces the functional Env expression on the surface of infected MT-4 cells.

Because the proteolytic processing of gp160 is absolutely required for activation of the fusogenic activity of Env, we next examined the effect of fangchinoline on gp160 processing. Env protein expression in cells transfected with the HIV-1 NL4-3 cDNA was analyzed by Western blot analysis using a polyclonal antibody directed against HIV-1 gp120. As shown in Fig. 5B, two distinct proteins (gp120 and gp160) were detected in the samples (upper panel). As the concentration of fangchinoline increased, the band intensity of gp120 decreased indicating a concentration-dependent inhibition of gp160 processing, which correlated with the inhibition of infectious virus production. As gp160 was a predominant protein detected in transfected cells (Fig. 5B, lane 1), we did not observe any obvious accumulation of uncleaved gp160 in fangchinoline treated cells (Fig. 5B, lane 2–4). To confirm that fangchinoline inhibits gp160 process, pulse–chase analysis was carried out to study the kinetics of gp160 processing in cells. pNL4-3 transfected 293T cells were labeled 1 hour and chased for 0, 2, 4, and 8 hours in the presence or absence of 10 µM fangchinoline. The results showed that newly synthesized gp160 was present in untreated cells and gradually decreased during the chase period. However, in the presence of fangchinoline, gp160 content remained unchanged up to 8 hours (Fig. 5C). Since the gp120 product was not observed under our experimental conditions, the decrease of the labeled gp160 could be a result of processing into gp120/gp41, incorporation into virus particles or degradation. However, as shown in Fig. 4B, the incorporation of gp160 into virus particles was not significantly affected by fangchinoline. Furthermore, the Western blots of whole cell lysates indicated that fangchinoline did not obviously alter the degradation of gp160 (Fig. 5B). Taken together, these results suggested that fangchinoline inhibits gp160 processing, rather than degradation or incorporation into virus particles.

## Discussion

Because target-based approaches are limited to developing novel treatments for validated targets, it is important to screen for inhibitors of novel targets in the context of a full viral replication cycle. In the present study, we screened more than 200 compounds, which were isolated from traditional Chinese medicinal herbs, using a full viral replication cycle assay resulting in the identification of fangchinoline as a novel anti-HIV-1 agent (Fig. 1). Our unpublished data showed that fangchinoline did not show an inhibitory effect on virus-induced CPE in influenza virus-infected MDCK cells or on HBV HBeAg or HBsAg expression in HepG2.117 cells, which indicates that the antiviral activity of fangchinoline may be specific to HIV-1. Although *in vitro* antiviral data indicated that the SI values were relatively low, the antiviral activity of fangchinoline was confirmed in a variety of assays and cannot be attributed to unspecific cell pathogenic effects. Of note, the further mode-of-action study showed that fangchinoline inhibits HIV-1 replication by impairing gp160 processing, a mechanism that has not been exploited by any commercially available drug for HIV-1 treatment.

Fangchinoline was demonstrated to have anti-HIV activity in full replication assays (Fig. 2 and Table 1) but not in the TZM-b1 assay (Table 2), indicating that it interferes with a process(es) after proviral transcription in the viral replication cycle. In the virus production/infectivity assay, fangchinoline reduced the infectivity of nascent virions and had no effect on viral release (Fig. 3A and 3B). After excluding the possibility that fangchinoline affects virion maturation (Fig. 3C), we found that the anti-HIV-1 activity of fangchinoline is Env dependent because the production of infectious VSV-G protein packaged HIV-1 virus was not affected (Fig. 4A). Further mode-of-action studies revealed that fangchinoline interferes with gp160 processing resulting in reduced cell-surface expression of functional Env and decreased Env incorporation into nascent virus (Fig. 4B and Fig. 5).

As mentioned above, a relatively low therapeutic index exists for fangchinline in the *in vitro* anti-HIV-1 assay, indicating the therapeutic utility in its current form is very limited. However, further structure-activity relationship study may help solve this problem. Fangchinoline has many naturally occurring analogues with a bisbenzylisoquinoline structure. Our unpublished data demonstrated that tetrandrine, a close related analogue of fangchinoline, exerted a similar anti-HIV-1 profile of fangchinoline. These analogues may serve in the structure-activity relationship study to uncover the scaffold responsible for the anti-HIV-1 activity of fangchinoline. Based on these data, chemical modification introduced into this compound may yield new derivative with reduced cytotoxicity and/or improved antiviral potency. However, until now, we can not determine from the chemical structure whether fangchinoline is a druggable compound, it is also possible that the chemical nature makes the compound can not move forward to further drug development.

Although all the results provide strong evidence that fangchinoline interrupts gp160 processing, the exact mechanism responsible for the observed inhibition of HIV production in fangchinoline treated cells requires additional study. In theory, several action mechanisms, either targeting host cells or the virus, can result in similar observations. Based on previously published data, fangchinoline may target host proprotein convertases involved in gp160 processing and inhibit their endoproteolytic activity. Fangchinoline was described to lower blood pressure as a non-specific Ca^2+^ channel antagonist [28]. Moreover, previous studies showed that Ca^2+^ is required for the processing of gp160 as Ca^2+^ depletion in the cellular compartment leads to a reduction in cleavage efficiency [29]. Thus, fangchinoline may impair the activity of these related enzymes by decreasing the concentration of Ca^2+^ in cells. However, it appears that the inhibition of the associated host proprotein-processing enzymes may be minimal, as fangchinoline did not exhibit activity against influenza viruses (data not shown). Similar to HIV-1, proteolytic activation of influenza virus hemagglutinin (HA) by host cell proteases is essential for viral infectivity. HA cleavage is likely mediated by one or more proprotein-processing subtilisin-related endoproteases of which furin is the leading candidate [30]. If fangchinoline impairs convertase activity by blocking Ca^2+^ channel, it should be active against both HIV-1 and influenza viruses. Thus, the anti-HIV-1 activity of fangchinoline may not be sufficiently explained by the inhibition of endoproteolytic activity of related host proprotein convertases. Of course, we cannot rule out the possibility that fangchinoline indirectly inhibits gp160 processing by disrupting oligosaccharide trimming or intracellular trafficking. As mentioned above, intracellular trafficking is essential for gp160 maturation and infectious virion production [6]. Megalomicin, a macrolide antibiotic has been shown to inhibit vesicular transport between medial-Golgi and trans-Golgi and consequently gp160 cleavage into its active forms [16]. Moreover, post-translational modifications of gp160 play a crucial role in its intracellular trafficking. Several compounds (castanospermine, deoxynojirmycin, and monensin) that inhibit glycosylation trimming, and subsequent gp160 trafficking and processing have been described [31], [32], [33]. However, such inhibitors target important host cell functions, thus, their clinical potential is limited due to safety concerns. If fangchinoline acts by any of these mechanisms mentioned above, its potential for further drug development will be limited.

Alternatively, fangchinoline may specifically target HIV-1 Env rather than host factors. In theory, fangchinoline may bind to HIV-1 Env and alter the natural conformation of gp160 in such a way that furin or other proprotein convertases are unable to access the cleavage site. In this case, the weak anti-HIV-1 activity should not be inherent in the profile of action, and fangchinoline may be used as a starting point for developing new HIV-1 therapeutic approaches. Based on further structure-activity relationship research, additional potent derivatives with lower toxicity may be developed. The successful development of Bevirimat (PA-457, DSB, MPC-4326) demonstrates the feasibility of utilizing natural products as the starting point for the development of new therapeutic approaches for HIV-1 infection [34]. Bevirimat is a first-in-class maturation inhibitor, and its development was based on a plant triterpene betulinic acid [35]. Although the original form of betulinic acid exhibited inhibitory activity against HIV-1 replication in H9 lymphocyte cells with a SI of only 9.3, its potent derivative 3-O-(30,30-dimethylsuccinyl) betulinic acid is currently in clinical trials [21].

In summary, our results presented in this study demonstrate that fangchinoline inhibits HIV-1 replication by interfering with gp160 proteolytic processing. Although the therapeutic utility in its current form is limited, fangchinoline may serve as a starting point for developing a new HIV-1 therapeutic approach or as a basic research tool for interrogating events during HIV Env maturation.

## References

[1] Carr A, Cooper DA (2000). Adverse effects of antiretroviral therapy.. The Lancet.

[2] Montessori V, Press N, Harris M, Akagi L, Montaner JSG (2004). Adverse effects of antiretroviral therapy for HIV infection.. Canadian Medical Association Journal.

[3] Cohen MS, Hellmann N, Levy JA, DeCock K, Lange J (2008). The spread, treatment, and prevention of HIV-1: evolution of a global pandemicJournal of Clinical Investigation.. The Journal of Clinical Investigation.

[4] Clavel F, Hance AJ (2004). HIV drug resistance.. New England Journal of Medicine.

[5] Checkley MA, Luttge BG, Freed EO (2011). HIV-1 envelope glycoprotein biosynthesis, trafficking, and incorporation.. Journal of Molecular Biology.

[6] Moulard M, Decroly E (2000). Maturation of HIV envelope glycoprotein precursors by cellular endoproteases.. Biochimica et Biophysica Acta (BBA) – Reviews on Biomembranes.

[7] McCune JM, Rabin LB, Feinberg MB, Lieberman M, Kosek JC (1988). Endoproteolytic cleavage of gp160 is required for the activation of human immunodeficiency virus.. Cell.

[8] Jiang Y, Liu X, De Clercq E (2011). New therapeutic approaches targeted at the late stages of the HIV-1 replication cycle.. Current Medicinal Chemistry.

[9] Sabatier JM, Mabrouk K, Moulard M, Rochat H, Van Rietschoten J (1996). Anti-HIV activity of multibranched peptide constructs derived either from the cleavage sequence or from the transmembrane domain (gp41) of the human immunodeficiency virus Type 1 envelope.. Virology.

[10] Barbouche R, Sabatier JM, Fenouillet E (1998). An anti-HIV peptide construct derived from the cleavage region of the Env precursor acts on Env fusogenicity through the presence of a functional cleavage sequence.. Virology.

[11] Barbouche R, Decroly E, Kieny MP, Fenouillet E (2000). An anti-human immunodeficiency virus multiple antigen peptide encompassing the cleavage region of the env precursor interferes with membrane susion at a post-CD4 binding step.. Virology.

[12] Cameron A, Appel J, Houghten RA, Lindberg I (2000). Polyarginines are potent furin inhibitors.. Journal of Biological Chemistry.

[13] Kibler KV, Miyazato A, Yedavalli VSRK, Dayton AI, Jacobs BL (2004). Polyarginine Inhibits gp160 processing by furin and suppresses productive human immunodeficiency virus type 1 infection.. Journal of Biological Chemistry.

[14] Adessi C, Soto C (2002). Converting a peptide into a drug: Strategies to improve stability and bioavailability.. Current Medicinal Chemistry.

[15] Thomas G (2002). Furin at the cutting edge: From protein traffic to embryogenesis and disease.. Nature Reviews: Molecular Cell Biology.

[16] José ES, Muñoz-Fernández MA, Alarcón B (1997). Megalomicin inhibits HIV-1 replication and interferes with gp160 processing.. Virology.

[17] Dewar RL, Vasudevachari MB, Natarajan V, Salzman NP (1989). Biosynthesis and processing of human immunodeficiency virus type 1 envelope glycoproteins: effects of monensin on glycosylation and transport.. Journal of Virology.

[18] Gruters RA, Neefjes JJ, Tersmette M, de Goede REY, Tulp A (1987). Interference with HIV-induced syncytium formation and viral infectivity by inhibitors of trimming glucosidase.. Nature.

[19] Blair WS, Cao J, Jackson L, Jimenez J, Peng Q (2007). Identification and characterization of UK-201844, a novel inhibitor that interferes with human immunodeficiency virus type 1 gp160 processing.. Antimicrobial Agents and Chemotherapy.

[20] Vlietinck AJ, De Bruyne T, Apers S, Pieters LA (1998). Plant-derived leading compounds for chemotherapy of human immunodeficiency virus (HIV) Infection.. Planta Medica 64: 97,109.

[21] Saklani A, Kutty SK (2008). Plant-derived compounds in clinical trials.. Drug Discovery Today.

[22] Miyake A, Ishida T, Yamagishi M, Hara T, Umezawa K (2010). Inhibition of active HIV-1 replication by NF-κB inhibitor DHMEQ.. Microbes and Infection.

[23] Wan H, Seth A, Rainen L, Fernandes H (2010). Coamplification of HIV-1 proviral DNA and viral RNA in assays used for quantification of HIV-1 RNA.. Journal of Clinical Microbiology.

[24] Platt EJ, Bilska M, Kozak SL, Kabat D, Montefiori DC (2009). Evidence that ecotropic murine leukemia virus contamination in TZM-bl Cells does not affect the outcome of neutralizing antibody assays with human immunodeficiency virus type 1.. Journal of Virology.

[25] Li F, Goila-Gaur R, Salzwedel K, Kilgore NR, Reddick M (2003). PA-457: A potent HIV inhibitor that disrupts core condensation by targeting a late step in Gag processing.. Proceedings of the National Academy of Sciences.

[26] Blair WS, Pickford C, Irving SL, Brown DG, Anderson M (2010). HIV capsid is a tractable target for small molecule therapeutic intervention.. PLoS Pathogens.

[27] Blair WS, Cao J, Fok-Seang J, Griffin P, Isaacson J (2009). New small-molecule inhibitor class targeting human immunodeficiency virus type 1 virion maturation.. Antimicrobial Agents and Chemotherapy.

[28] Kim HS, Zhang YH, Oh KW, Ahn HY (1997). Vasodilating and hypotensive effects of fangchinoline and tetrandrine on the rat aorta and the stroke-prone spontaneously hypertensive rat.. Journal of Ethnopharmacology.

[29] Moulard M, Montagnier L, Bahraoui E (1994). Effects of calcium ions on proteolytic processing of HIV-1 gp160 precursor and on cell fusion.. FEBS Letters.

[30] Alexander DJ (2007). An overview of the epidemiology of avian influenza.. Vaccine.

[31] Pal R, Gallo RC, Sarngadharan MG (1988). Processing of the structural proteins of human immunodeficiency virus type 1 in the presence of monensin and cerulenin.. Proceedings of the National Academy of Sciences.

[32] Pal R, Hoke GM, Sarngadharan MG (1989). Role of oligosaccharides in the processing and maturation of envelope glycoproteins of human immunodeficiency virus type 1.. Proceedings of the National Academy of Sciences.

[33] Walker BD, Kowalski M, Goh WC, Kozarsky K, Krieger M (1987). Inhibition of human immunodeficiency virus syncytium formation and virus replication by castanospermine.. Proceedings of the National Academy of Sciences.

[34] Aiken C, Chen CH (2005). Betulinic acid derivatives as HIV-1 antivirals.. Trends in Molecular Medicine.

[35] Fujioka T, Kashiwada Y, Kilkuskie RE, Cosentino LM, Ballas LM (1994). Anti-AIDS agents, 11. Betulinic acid and platanic acid as anti-HIV principles from syzigium claviflorum, and the anti-HIV activity of structurally related triterpenoids.. Journal of Natural Products.

